# Applications and prospects of cryo-electron tomography in drug discovery and understanding disease

**DOI:** 10.1016/j.sbi.2026.103283

**Published:** 2026-05-14

**Authors:** Camila M. Clemente, Juan-Carlos Mobarec, Tanmay A. M. Bharat

**Affiliations:** 1 Structural Studies Division, MRC Laboratory of Molecular Biology, Francis Crick Avenue, Cambridge CB2 0QH, United Kingdom; 2 Discovery Sciences, Protein Structure and Biophysics, R&D, Astra-Zeneca, Cambridge, United Kingdom

## Abstract

Cryo-electron tomography (cryo-ET) is emerging as a transformative tool for structural biology. Unlike methods based on purified molecules, cryo-ET enables visualisation of macromolecules directly within intact cells and tissues, preserving their native interactions and physiological context. By providing high-resolution views of healthy and diseased cells, cryo-ET offers a powerful means to understand infection and disease mechanisms. Moreover, cryo-ET combined with subtomogram averaging can resolve macromolecular structures beyond 3 Å resolution, making it a promising approach for computational drug discovery. This article highlights the recent contributions from cryo-ET in understanding human disease and examines future perspectives of this rapidly evolving technique in structure-based drug discovery. We propose a roadmap for the developments required for its widespread adoption in pharmacological research.

## The evolving contribution of structural biology in understanding disease and assisting drug discovery

Understanding how target molecules interact, how drugs reshape their conformations, and how these molecular changes affect cellular function is fundamental for therapeutic innovation. Due to these reasons, structure-based approaches are highly synergistic with other techniques and can significantly accelerate drug discovery. Over the years, advances in X-ray crystallography and nuclear magnetic resonance (NMR) have delivered transformative insights into the structures of drug targets, from ion channels to G-protein-coupled receptors (GPCRs) [1], and many present-day medicines trace their origins to such pioneering structural studies [2]. More recently, in the last decade, electron cryomicroscopy single-particle analysis (cryo-EM SPA) has provided a quantum leap in structural studies by enabling atomic-resolution structures of purified macromolecular complexes [3]. This progress has been driven by advances in microscope instrumentation [4], the development of direct electron detectors [5], and powerful image-processing algorithms [6,7].

Alongside these experimental approaches, artificial intelligence (AI)-driven methods have recently transformed structural biology. Tools such as AlphaFold2 [8] and RoseTTAFold [9] have demonstrated that protein structures could be predicted with near-experimental accuracy from sequence alone. More recently, Alpha-Fold3 [10] and RoseTTAFold All-Atom [11] have extended these capabilities to predict full biomolecular assemblies―including protein—ligand, protein—nucleic acid, and multi-component complexes―greatly expanding their utility for structure-based drug discovery. Notably, these computational models can serve as improved starting references for experimental pipelines, providing complementary information.

The impact of cryo-EM SPA has improved drug-discovery pipelines by providing detailed insights into drug—target interactions that inform rational design. Landmark examples from pathogens include the rapid structural characterisation of the SARS-CoV-2 spike protein, which directly guided the development of vaccines [12,13] and the structure-guided design of antibodies targeting the type 3 secretion system (T3SS) virulence factor PcrV from pathogenic *Pseudomonas aeruginosa* bacteria [14]. Cryo-EM has also revolutionised structural studies of GPCRs [15], which represent nearly 36% of Food and Drug Administration (FDA)-approved drug targets [16] as well as amyloids associated with neurodegenerative diseases [17]. Such achievements underscore the central role of SPA in structure-guided therapeutic development and in the understanding of disease. Future developments in cryo-EM technology [18,19] are expected to further expand these insights.

However, SPA has limitations in structure-based drug discovery, particularly when capturing ligand binding and mechanisms of action in their native cellular or tissue context [20]. This is because SPA typically relies on biochemical isolation and purification of target molecules, which may adopt conformations different from those in cells, leading to the possibility of missing interactions that only occur in their native context [21,22]. This knowledge gap between the fine molecular details and biological complexity at the cellular and tissue level defines the next challenge for the field. Can structural biology be extended directly into the complex native multicellular environment of tissues?

## From reductionist models to cellular landscapes: an overview of cryo-ET

Toward this ambitious goal, cryo-electron tomography or electron cryotomography (cryo-ET) is emerging as a powerful technique, offering *in situ* visualisation of macromolecules within their native structural context, from reconstituted assemblies that preserve relevant molecular interactions or directly from cells and tissues, thus avoiding the conformational perturbations introduced by biochemical isolation [20,23]. Throughout this review, we use *in situ* in this broader sense, encompassing structures determined within intact cellular environments (*in cellulo*) as well as from nearly native reconstituted assemblies such as virus-like particles.

**Figure 1:**
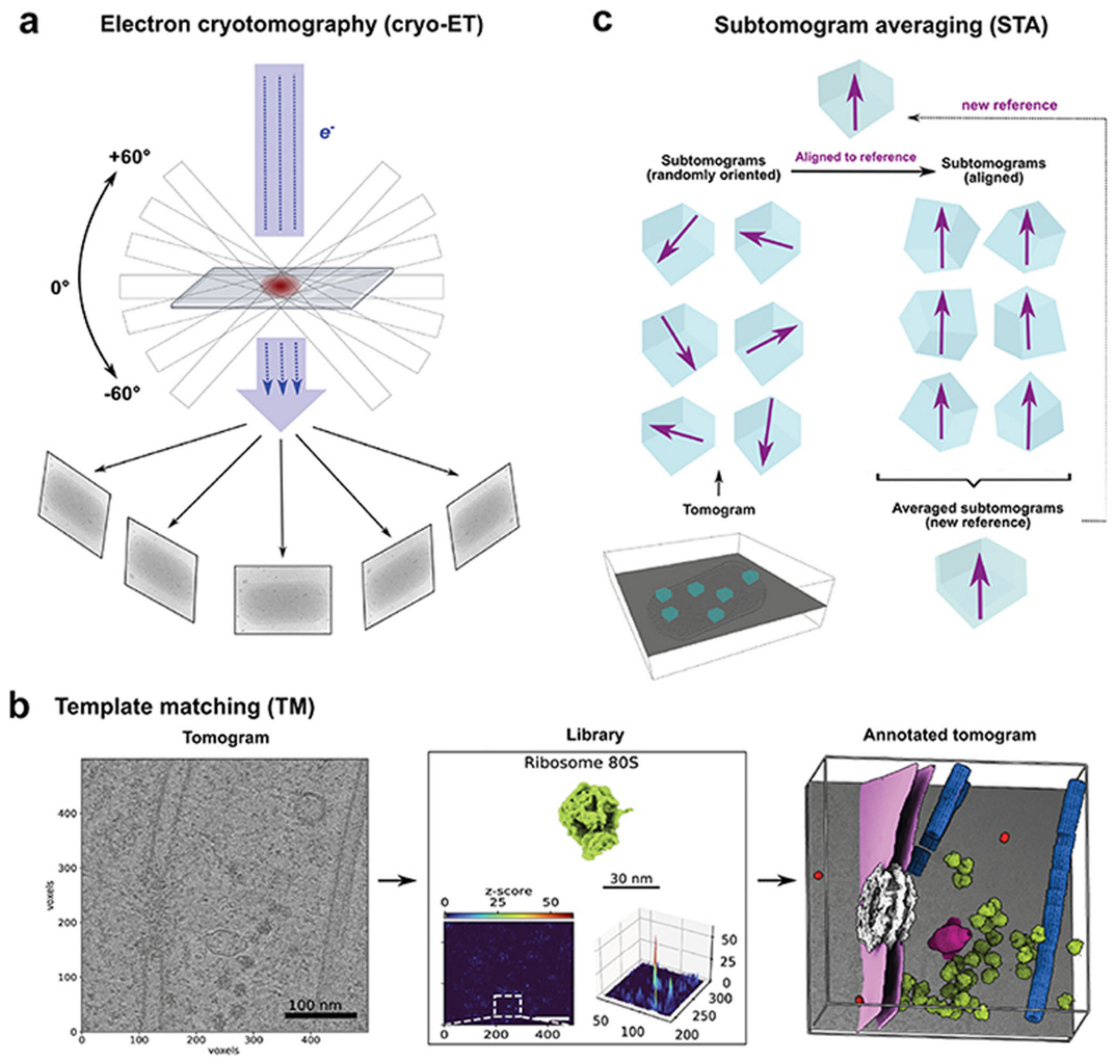
Schematic of cryo-ET, TM, and STA. (**a**) In cryo-ET, the vitrified sample is incrementally tilted within a typical range between ±60° relative to the electron beam, and images are acquired on a direct electron detector. The resulting tilt series is computationally aligned and back-projected to reconstruct a three-dimensional volume known as the tomogram. (**b**) TM is performed by comparing the tomographic density with known atomic structures called templates to locate their positions within the tomogram using an appropriate figure of merit, producing 3D localisation maps for visualisation and analysis of the spatial organisation of complexes (adapted from Ref. [37]). (**c**) In STA, subtomograms (or sub-volumes) containing a copy of the macromolecule of interest are extracted from tomograms (or from the tilt images in SPT). These extracted subtomograms are then iteratively aligned to a reference and averaged to generate a higher-resolution density map, opening the possibility of solving the atomic structures of macromolecules inside cells. cryo-ET, cryo-electron tomography; STA, subtomogram averaging; TM, template matching.

Cryo-ET involves acquiring multiple ‘tilted’ images of the vitrified biological specimens and computationally combining them into a three-dimensional (3D) tomogram that shows the internal arrangement of the specimen at a high-resolution (usually 3—4 nm) (Figure 1a). Cryo-ET may be combined with a complementary computational technique called template matching (TM), which is used to detect specific macromolecular complexes in noisy tomograms by comparing them to reference atomic structures (Figure 1b), effectively mapping macromolecules *in situ* [24]. Recent advances in TM, including *de novo* template generation and deep-learning frameworks such as DeepFinder [25], DeePiCt [26], TomoTwin [27] and 2DTM [28], have markedly improved both the accuracy and speed of macromolecular identification, leading to a deeper understanding of the interior arrangement of cells.

Once the macromolecules of interest have been identified and localised with TM, they can be further analysed with subtomogram averaging (STA) or single-particle tomography (SPT), which require the extraction and averaging of repeated macromolecular densities from tomograms (Figure 1c). STA (or SPT) can supply macromolecular reconstructions typically ranging from sub-nanometre to a few nanometres in resolution, depending on the abundance and structural homogeneity of the target [29—34]. Modern STA software packages have enabled near-atomic resolution reconstructions (3—4 Å-resolution) directly within the native cellular context [30,31], although achieving resolutions sufficient for *ab initio* atomic model building remains the exception rather than the rule [33—35].

For samples exceeding ~300 nm in thickness, such as intact tissues or multicellular assemblies, cryo-focused ion beam (cryo-FIB) milling can be applied prior to cryo-ET [20]. Cryo-FIB is used to gradually ablate cellular or tissue material, generating thin electron-transparent lamellae from specimen regions that would otherwise be too thick for cryo-EM due to increased inelastic scattering [36], enabling high-resolution imaging of cellular interiors while preserving the native environment. Cryo-ET, with these associated tools, represents a paradigm shift in structural biology with direct implications for understanding disease processes and accelerating drug discovery. In the remaining sections of this article, we will highlight recent contributions from cryo-ET, with a view to what the technique could offer in the coming years in this area.

## Visualising disease pathology *in situ*

Cryo-ET can reveal pathological signatures of diseased cells and tissues at the molecular level, which remain hidden to other techniques and can often offer novel insights even with when reconstructed tomograms of diseased cells are visually inspected. Combined with TM and STA, cryo-ETallows researchers to compare the locations, structures, and conformations macromolecular assemblies between healthy and diseased cells. The examples highlighted below, although not exhaustive, showcase some recent applications of cryo-ET combined with TM and STA that have advanced our understanding of disease mechanisms at the molecular level.

**Figure 2:**
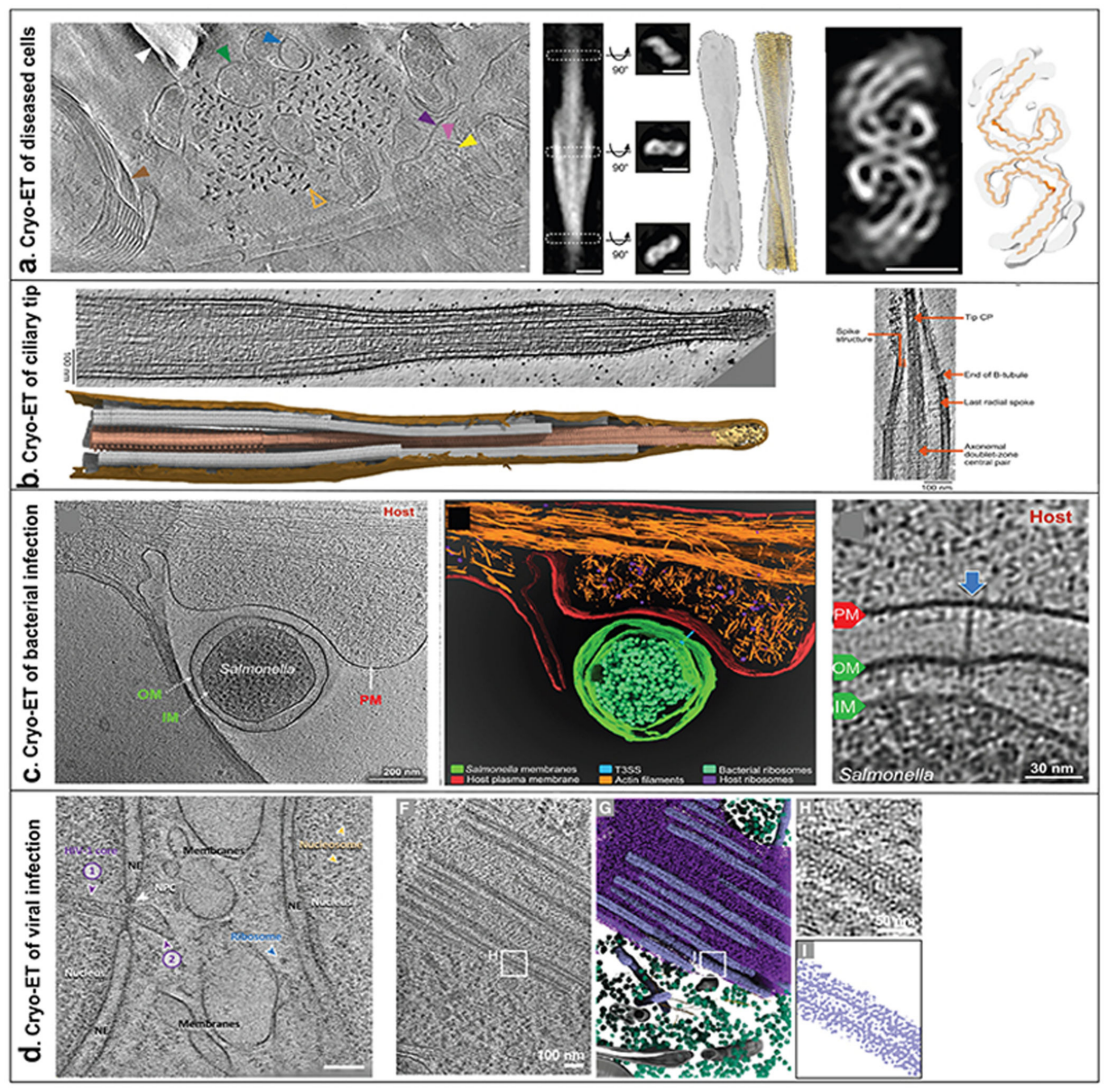
Cryo-ET derived structural insights into disease and infection. (**a**) Alzheimer’s disease. (First panel) Tomographic slice of Alzheimer’s brain showing extracellular tau filaments and surrounding organelles, scale bar 10 nm. (Second panel) STA reveals paired C-shaped protofilaments. (Third Panel) Helical STA resolves filament architecture at ~8.7 Å ([40]). (**b**) Ciliopathies. (First panel) Tomogram slice of a representative ciliary tip and 3D model of the same cilium generated with the subtomogram averages described in this study. (Second panel) Representative slice of a demembranated cilium (adapted from Ref. [43]). (**c**) Bacteria infection. (First panel) Tomographic slice of a *Salmonella* minicell attached to a host cell. (Second panel) 3D segmentation shows membranes, T3SS injectisomes, actin, and ribosomes. (Third panel) Tomographic slice showing T3SS needles contacting the host plasma membrane and bending it without penetration (adapted from Ref. [47]). (**d**) Viral infection. (First panel). A representative tomographic slice of a tomogram showing two successive HIV-1 cores at the same nuclear pore complex (NPC), indicated by purple arrowheads and numbered. The NPC, ribosome, prominent nucleosomes, the nucleus, nuclear envelope (NE), and membranes are annotated. Scale bar, 100 nm. (adapted from Ref. [49]). (Second panel). Viral factories inside infected cells show rigid bundles of assembled nucleocapsids, with ribosomes invading the factory space (adapted from Ref. [50]). cryo-ET, cryo-electron tomography; STA, subtomogram averaging.

By enabling the visualisation of neurons and neural tissue, cryo-ET has provided critical insights into the molecular alterations underlying neurodegenerative disease progression. Early work on neuronal proteostasis that combined cryo-ET, TM, and STA demonstrated that only ~20% of proteasomes are active in resting neurons, thereby uncovering a reserve pool of inactive complexes that could be mobilised under stress conditions [38]. In another study on Parkinson’s disease, α-synuclein inclusions were resolved in focused-ion beam-milled neurons, which showed fibrillar aggregates interspersed with organelles, demonstrating that short fibrils nucleate and promote aggregate growth [39]. In Alzheimer’s disease, cryo-ET and STA resolved β-amyloid plaques and distinct tau filament conformations at subnanometre resolution, providing mechanistic insight into pathogenic aggregation and providing clues to potential drug-binding sites [40] (Figure 2a).

In the same vein, cryo-ET with STA has provided essential insights into the molecular architecture of cilia and their disruption in ciliopathies, such as retinal degeneration. It has revealed the structural remodelling of intraflagellar transport trains during cargo delivery [41], the conformational dynamics of dynein motors driving ciliary beating [42], and the stabilising role of tip-associated proteins that regulate axoneme integrity and length [43] (Figure 2b). Moreover, cryo-ET resolved the native organisation of rootlet filaments composed of rootletin and associated proteins, which anchor cilia to the cell body. Mutations in these components compromise ciliary stability, leading to human ciliopathies such as retinal degeneration [44]. Finally, in the context of muscle disorders, cryo-ET has elucidated how nebulin acts as a molecular ruler stabilising thin filaments, offering key mechanistic insights that inform the development of therapeutic strategies for muscle disorders [45].

## Cryo-electron tomography studies of infection

Cryo-ET has also shed light on host—pathogen interactions in bacterial, viral, and parasitic infections. For the human pathogen *Helicobacter pylori*, cryo-ET revealed the molecular architecture of the cag type 4 secretion system (T4SS) *in vivo*, showing how this nanomachine induces membranous tubes with lateral ports, when the bacterium encounters host cells. These tubes likely serve as channels for delivering virulence factors directly into host cells. Cryo-ET imaging highlighted how *H. pylori* remodels host membranes to establish infection [46]. Similarly, in cells infected with *Salmonella enterica*, cryo-ET visualised the type III secretion system (T3SS) injectisome in direct contact with host membranes, capturing the *in situ* structure of the translocon complex. This translocon allows the bacterium to inject virulence factors into host cells, manipulating cellular processes to promote infection. The close association between the injectisome and host membrane also triggers remodelling of the membrane, which likely facilitates bacterial entry and enhances the pathogen’s ability to cause disease [47] (Figure 2c).

For viral pathogens, recent correlative cryogenic light and electron microscopy (cryo-CLEM) of HIV-1 infected cells enabled the visualisation of intact, mature, cone-shaped capsids crossing the nuclear pore complex, reshaping current models of viral uncoating [48]. Even more recently, an integrated correlative workflow combining cryo-FIB milling and cryo-ET enabled precise *in situ* visualisation of HIV-1 cores at distinct stages of nuclear import across nuclear pores, capturing both docked and translocating capsids *in situ* [49] (Figure 2d). For another important human pathogen Ebola virus, cryo-ET captured multiple stages of viral assembly and budding at the plasma membrane, providing structural snapshots of the viral replication cycle in its native cellular context (Figure 2d) [50]. Complementary studies of Ebola virus replication factories further illuminated the intracellular assembly of the viral nucleocapsid, revealing previously unresolved nucleocapsid interactions with the viral matrix [51].

In parasitic organisms, cryo-ET of *Plasmodium falciparum*, the parasite responsible for malaria, has revealed stage-specific differences in microtubule organization throughout its life cycle [52]. Moreover, recent *in situ* analyses of *P. falciparum* merozoites showed that the apicoplast, an essential plastid-like organelle required for key metabolic pathways such as isoprenoid biosynthesis, is enclosed by four membranes [53]. From a drug-design perspective, knowing that the apicoplast lumen is separated from the cytosol by multiple membranes is crucial to know whether candidate drugs would need to cross these barriers to reach their target or whether they could instead target cytosolic factors essential for apicoplast function and maintenance.

## High-resolution cryo-electron tomography of pharmacologically relevant targets

**Figure 3:**
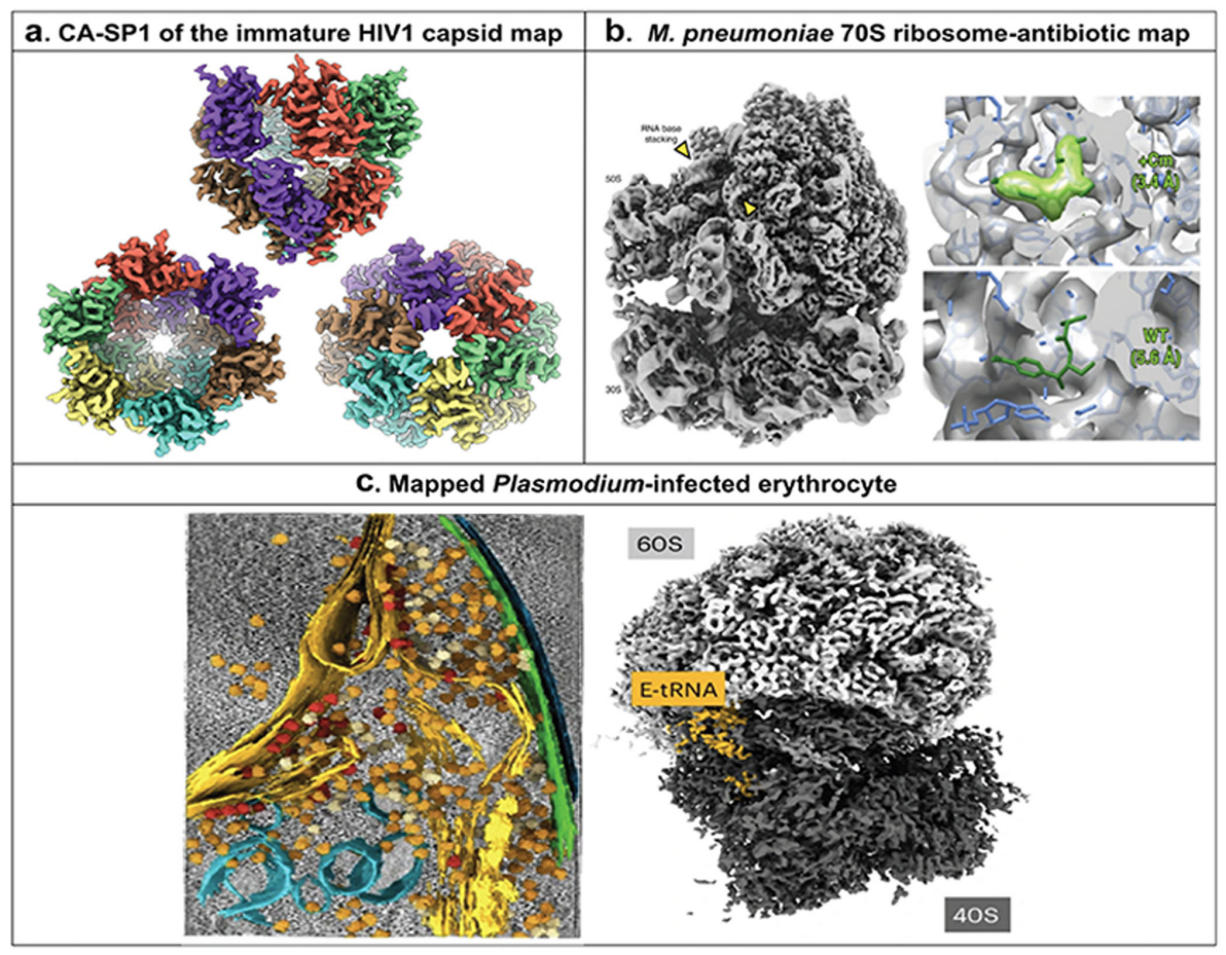
Cryo-ET and STA of pathogen macromolecular complexes. (**a**) STA map of the immature HIV-1 capsid (CA-SP1) at 3.0 Å resolution, shown in multiple views, adapted from Ref. [30]). (**b**) *M. pneumoniae* 70S ribosome bound to chloramphenicol. STA of chloramphenicol-treated cells enabled visualisation of the antibiotic binding site inside intact cells [56]. (**c**) Segmented tomograms of parasite-infected red blood cells, with STA reconstruction of the Pf-80S ribosome (60S–40S) showing bound E-site tRNA (orange) at 4.1 Å resolution [58]. cryo-ET, cryo-electron tomography; STA, subtomogram averaging.

A landmark study that presented a *de novo* atomic model built directly from STA, using a highly optimised workflow reported a 3.9 Å resolution structure of the HIV-1 capsid in immature virus-like particles treated with the virus maturation inhibitor bevirimat. The structure revealed a six-helix bundle encompassing the CA (capsid)—SP1 (spacer peptide 1) cleavage site, which is inaccessible to protease in the immature lattice. Bevirimat was proposed to prevent viral maturation by stabilising this bundle, thereby blocking proteolytic cleavage at the CA—SP1 [54]. Importantly, this work provided an *in situ* structure of the immature capsid—inhibitor complex, extending earlier crystal structures of isolated capsid domains into the native viral lattice. Since this study, new STA software packages such as RELION-5 and emClarity have been developed that report similar or better STA reconstructions of the same biological specimen [30,55] (Figure 3a).

Next, high-resolution in-cell STA was made possible using the M software framework, which uses a SPT (or a constrained single-particle EM) approach for refinement. By accurately correcting for radiation-induced sample deformation at the particle and tilt-image level, chloramphenicol bound 70S ribosomes inside intact *Mycoplasma pneumoniae* cells were visualised at 3.5 Å resolution. This result provided compelling evidence that cryo-ET can provide relevant structural detail *in situ*, directly visualising the antibiotic binding pocket within the ribosome in its native cellular context [56] (Figure 3b). Notably, Xu et al. (2025) further improved the resolution to 3.0 Å, enabling more detailed visualisation of chloramphenicol coordination within the peptidyl transferase centre [57].

Most recently, *in situ* cryo-ET and STA of *P. falciparum*-infected erythrocytes, were used to resolve several native intermediates of the ribosome elongation cycle, showing how the translation inhibitor cabamiquine (CBQ) perturbs elongation factor binding and ribosome biogenesis. This study provides a molecular level visualisation of drug-induced changes in malaria parasites within their native cellular environment [58] (Figure 3c).

Together, these studies illustrate how methodological innovations in cryo-ET, TM, and STA―both in hardware and computational pipelines―are enabling *in situ* visualisation of crucial drug—target interactions at resolutions previously thought to be exclusive to SPA and other structural techniques. Such *in situ* structures show the ‘real’ cellular conformations of macromolecules, which should accelerate structure-based drug discovery.

## From structural limitations to drug discovery opportunities

Despite recent progress, cryo-ET still faces fundamental limitations that restrict its broader use. The first limitation is related to sample preparation, which is relatively cumbersome, involving FIB-milling and long data-collection times, when compared to SPA [20]. Secondly, the intrinsically low signal-to-noise ratio makes it challenging to achieve resolutions comparable to SPA. Furthermore, obtaining high-resolution structures also relies on the natural abundance of target proteins within cells, since many copies of the target macromolecules are required for STA. These limitations affect both the achievable structural detail and the practical use of the data for pharmacological studies.

Recent developments are steadily overcoming many of the sample-preparation limitations, by automating many of the cryo-ET-related workflows [59]. The use of hardware improvements such as the laser phase plate could offer a step change in tomogram quality and is paralleled by advances in FIB milling that markedly improve sample preparation and lamella quality [19,60]. Moreover, recent innovations in software for data analysis have significantly improved both TM and boosted STA resolution, bringing the technique closer to becoming a routine structural biology approach, reducing manual work from months to a few days [22].

Combining cryo-ET with complementary approaches promises to increase its impact in the field, for example, allowing the localisation of small molecules in cells and tissues by correlative mass spectrometry imaging [61], or by allowing the localisation of drug targets by CLEM [62]. Equally, integrating molecular dynamics simulations [63] and AI-powered macromolecular modelling with cryo-ET has the potential to deliver a step-change in the field. Indeed, AlphaFold-predicted models are already being fitted into cryo-ET maps where experimentally-determined structures are unavailable, enabling protein identification and model building at intermediate resolutions [64,65].

In summary, we believe that cryo-ET is advancing from a specialised structural biology method to a technology with direct pharmaceutical impact. Its ability to capture macromolecular complexes, conformational states, and even small-molecule interactions within the native environment is beginning to reshape how druggable targets are identified and validated. Academic and industrial initiatives are already capitalising on these capabilities, with fully automated pipelines now streamlining both data acquisition and analysis. Looking forward, continued improvements in throughput, resolution, and integration with complementary computational and imaging approaches will be critical for the next revolution in structural pharmacology.
